# Health apps targeting children with overweight—a protocol for a systematic review with meta-analysis and Trial Sequential Analysis of randomised clinical trials

**DOI:** 10.1186/s13643-020-1269-0

**Published:** 2020-02-11

**Authors:** Rajeeb Rashid, Paolo Perego, Laura Condon, Janus Christian Jakobsen, Jane Lindschou, Christian Gluud, Giuseppe Andreoni, Inge Lissau

**Affiliations:** 1 Paediatric Unit, Department of Child Health, University of Edinburgh, St John’s Hospital, Livingston, EH54 6PP UK; 2 Department of Design, Politecnico di Milano, via Durando 38/a, 20158 Milan, Italy; 3 School of Medicine, University of Nottingham, Medical School, Nottingham, NG7 2RD UK; 4 Copenhagen Trial Unit, Centre for Clinical Intervention Research, Rigshospitalet, Copenhagen University Hospital, Blegdamsvej 9, 2100 Copenhagen Ø, Denmark; 5 Department of Cardiology, Holbæk Hospital, Holbæk, Denmark; 6 Clinical Research Centre, University Hospital Copenhagen, Kettegard Alle 30, 2650 Hvidovre, Denmark

**Keywords:** Obesity, Overweight, Children, Adolescents, Smartphone app, Health app, mHealth app, Management, Intervention, Treatment

## Abstract

**Background:**

The prevalence of overweight is increasing worldwide in children. Multi-component interventions incorporating diet, physical activity, and behavioural change have been shown to reduce body mass index (BMI). Whilst many children have their own smartphone, the clinical effects of using smartphone applications (apps) for overweight are unknown. This systematic review aims to ascertain the effects of mHealth apps in children with overweight.

**Methods:**

We will include randomised clinical trials irrespective of publication type, year, status, or language. Children between 0 and 18 years with overweight will be included. We will compare apps targeting overweight versus sham app, no app, or usual intervention. No distinction about operative system will be considered (i.e. Android, iOS, and Window Mobile will be included). The following databases will be searched: The Cochrane Library, Excerpta Medica database (Embase), PsycINFO, PubMed, IEEE Explore, Web of Science, CINAHL, and LILACS. Primary outcomes will be body weight, quality of life, and serious adverse event. Secondary outcomes will be self-efficacy, anxiety, depression, and adverse event not considered serious. Trial inclusion, data extraction, and bias risk assessment will be conducted independently by at least two authors. We will assess risk of bias through eight domains and control risks of random errors with Trial Sequential Analysis. The quality of the evidence will be assessed using Grading of Recommendations Assessment, Development and Evaluation Tool (GRADE).

**Discussion:**

We will provide evidence of the beneficial and harmful effects of smartphone apps for children with overweight and highlight any gaps in the evidence in order to shape future potential interventions. By only including randomised clinical trials, we know that we bias our review towards benefits.

**Systematic review registration:**

PROSPERO CRD42019120210

## Background

The prevalence of overweight is increasing worldwide both among children and adults [1–3]. Despite significant resources being spent on reversing this trend, the rates of paediatric overweight have risen worldwide over the last few decades with an estimated 124 million obese children and adolescents [4, 5]. This has also been associated with widening health inequality, as the prevalence of obese children is higher in areas of social deprivation [6]. Recent preliminary data by WHO European Childhood Obesity Surveillance Initiative have shown a decrease in childhood obesity prevalence over 10 years in Greece, Italy, Portugal, and Slovenia but acknowledges that changes are unequally distributed in all populations, again highlighting potential health inequality [7]. International Task Force of Obesity produced age- and sex-specific cut-off for the definition of overweight and obesity in children [8]. Throughout this paper, we will use the term overweight for all children with overweight including all levels of obesity.

Children with overweight will potentially have both short- and long-term comorbidities on cardiovascular disease, insulin resistance, type 2 diabetes, metabolic syndrome, and cancer (endometrial, breast, and colon). These result in a significant burden on the individual as well as health services across the world [5–9]. The severity of these comorbidities typically increases with the severity of overweight [10, 11] whilst mental health sequelae such as poor self-esteem, anxiety, and depression may result in bullying, discrimination, and long-term socioeconomic disadvantages [12–14].

### Mobile applications to support health (mHealth)

**Fig. 1:**
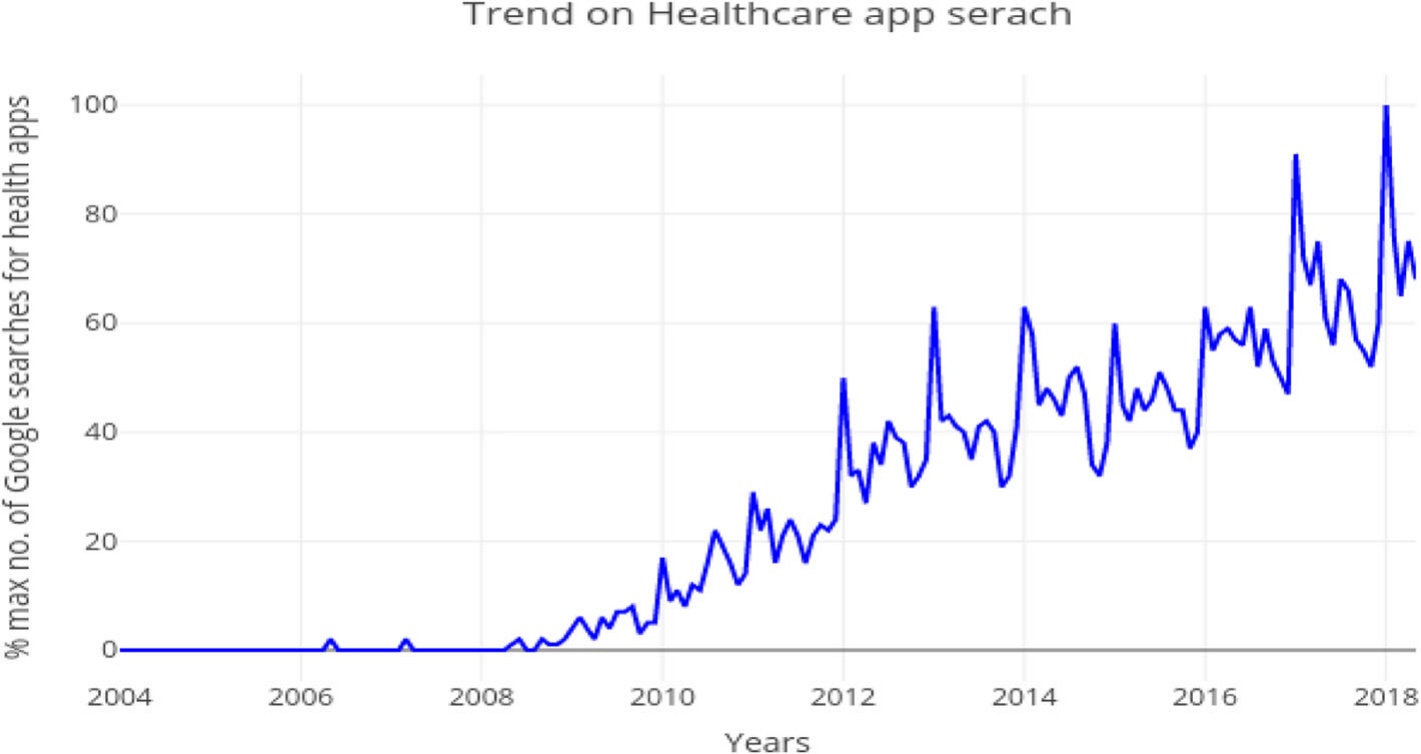
Trends of healthcare app searches on the Google Play app store since 2004 (mHealth phases with 100% being maximal number of Google searches for health apps)

During recent years, there has been an exponential global growth in Internet-connected devices such as smartphones for real-time communication, sharing of data, and running of multimedia software applications (apps). mHealth apps are software programs designed to support a healthy lifestyle and are among those most searched for and downloaded (Fig. 1) [15].

Children are a major consumer group for apps and subsequently present an opportunity to target the management of overweight in this population [16–19]. Apps thus represent a potentially effective medium for monitoring health parameters, interacting with individuals, and disseminating lifestyle interventions. However, paradoxically, apps can create an environment of social isolation, addiction, and anxiety through peer pressure and elevated sedentary activity through increased screen time [20–23].

Studies using mHealth have either used standalone apps or multi-component programmes which combine apps with direct sessions between the child and teacher/clinician. Whilst many studies targeted healthy behaviours, only a few formally incorporated behavioural change theory in achieving these goals [24, 25]. These apps were able to develop constructs based on self-determination or social cognitive theory to provide goal setting for nutrition, physical activity and screen time, tailored motivational messages, action planning, and reward systems. Such apps have the potential to support children with overweight to lose weight by supporting and strengthening their self-regulatory capacities [24, 26].

### Interventions in children with overweight

Cochrane reviews from 2017 on interventions for overweight in younger children (6–11 years) and older children (12–17 years) highlighted the paucity of good quality trials on multi-component interventions incorporating diet, physical activity, and behaviour change**.** Few studies looked at changes in quality of life with none showing any improvement in children post intervention and only a moderate improvement in older children, albeit in trials with a low quality of evidence [27, 28].

### Why is it important to do this review?

The increasing availability of smartphones for children and families across all socio-economic groupings may enable the use of apps to deliver, promote, and sustain multi-component interventions which could lead to long-term improvements in health [19]. Apps could create a multi-domain and customisable approach by leveraging the personal interests and motivational dimensions to provide long-term efficacy. Only a few previous reviews have focused on smartphone apps interventions in children with overweight [29]. Whilst previous reviews have commented upon the significant risk of bias in many studies, there has not been a consistency in including control of bias or assessing the quality of evidence with the Grading of Recommendations Assessment, Development and Evaluation Tool (GRADE) [30–37]. Our protocol thus aims to outline an up to date systematic review focussed on health apps in children with overweight, searching multiple databases, analysing a range of anthropometric and psychosocial outcomes and utilising GRADE and TSA methodology to assess risk of bias and type I and II errors.

#### Objective

The objective of this review is to assess the benefits and harms of mHealth apps targeting children with overweight versus sham app, no app, or usual intervention in children with overweight.

In particular, we will analyse the results at three levels:

- General outcomes of intervention apps regardless of their specific strategy; no distinction about operational system will be considered (i.e. Android, iOS, and Window Mobile will be included).
- Specific analysis according to the type of structured intervention promoted by the app: on the increase of physical activity, on nutrition, on psychotherapy and education, or in an integrated approach.
- Specific analysis according to the age range of participants: pre-school age (0–5 years), primary school age (younger children aged 6–11 years), secondary school age (older children aged 12 and below 18 years).

## Methods/design

### Eligibility criteria

#### Types of studies

Types of studies include randomised clinical trials irrespective of language, publication status, publication type, or publication year. Eligible studies which are not published in English will be translated using Google translate. By focusing on randomised clinical trials, we are aware that we focus on benefits and overlook harms. In case we find benefits of apps, systematic reviews on harms in observational studies need to be conducted.

#### Types of participants

Types of participants include all children who are overweight, up to 18 years of age. Children with associated co-morbidities, either physical or psychological secondary to overweight will be included.

#### Types of intervention

Types of intervention include all smartphone apps for intervention in children with overweight, independent of operating system and hardware platform. The control intervention can be a sham app, no intervention, or any current non-app intervention provided. There is no restriction for the duration of the intervention. Cointerventions are allowed if administered equally in the comparison groups.

#### Grey literature

There are many definitions of grey literature, but it is usually understood to mean literature that is not formally published in sources such as books or journal articles [38]. Conference abstracts and other grey literature have been shown to be sources of approximately 10% of the studies referenced in Cochrane reviews [39]. In a recently updated Cochrane methodology review, all five studies reviewed showed that published trials showed an overall greater treatment effect than grey literature trials [40]. Thus, failure to identify trials reported in conference proceedings and other grey literature might result in bias and affect the results of a systematic review.

### Outcomes

One of the most commonly used outcomes to compare results from intervention studies is the BMI Z-score. *Z*-scores are closely related to centiles and indicate the number of standard deviations the child’s measurement lies above or below the mean or median reference value [41]. Similarly, the increasing use of equipment such as bioimpedance and dual energy X-ray absorptiometry (DEXA) have provided information on fat mass and muscle mass in kilogrammes which are also well understood by families and can provide a useful measure over time [42, 43]. In addition, a serious adverse event will be defined as any untoward medical occurrence that results in disordered eating, significant or persistent morbidity, requires psychological or psychiatric treatment, hospitalisation or prolongation of existing hospitalisation.

We will assess all outcomes at two time points:

- End of intervention—primary time point of interest
- Maximum follow-up

Primary outcomes

- Body weight measured in kilogrammes
- Quality of life as measured by any scale that has been validated for use in the target population [44]
- Proportion of participants with at least one serious adverse event [45]

Secondary outcomes

- BMI *z*-score
- Self-efficacy as measured by a scale validated for use in children
- Anxiety
- Depression
- Proportion of participants with at least one adverse event not considered serious

Exploratory outcomes

- Body fat (percentage) measured by bioimpedance or DEXA, good correlation having been shown between total body fat percentage and bioimpedance DEXA (*r* = 0.87, *P* < 0.001) [42, 43]
- Muscle mass (kilogrammes) via bioimpedance or DEXA [42, 43]
- Individual serious and non-serious adverse events

### Search strategy

We will search the following databases: Cochrane Library; MEDLINE; Excerpta Medica database (Embase); PsychINFO, IEEE Explore, Web of Science (SCI-Expended, SSCI, A&HCI, CPCI-S, CPCI-SSH, ESCI, CCR-EXPANDED, IC), CINAHL, LILACS, OpenSIGLE, and Healthcare Management Information Consortium (HMIC).

In addition, we will search the following online resources: ClinicalTrials.gov (http://www.clinicaltrials.gov/), Google Scholar (https://scholar.google.com/), European Medicine Agency (https:// www.ema.europa.eu/ema/), United States Food and Drug Administration (www.fda.gov), Medicines and Healthcare Products Regulatory Agency (https://www.gov.uk/government/organisations/medicines-and-healthcare-products-regulatory-agency), The World Health Organization (www.who.int/), Global Obesity Forum (previously International Association for the study of Obesity) (www.iaso.org), European Association for the study of Obesity (EASO) (easo.org), and ICTRP Search Portal.

Finally, keywords used in search strategies will be the following: Obesity, Overweight, Smartphone apps, Health apps, mHealth apps, Body Mass Index, Weight Gain, Weight Loss, and Hyperphagia (Additional file 1).

### Data collection process

#### Selection of studies

The review will follow the recommendations in the Cochrane Handbook for Systematic Reviews of Interventions and according to Keus and colleagues and Jakobsen and colleagues [46–49]. The analyses will be performed using Review Manager [50] and Trial Sequential Analysis programme [51]. Two authors (RR and PP) will independently screen titles and abstracts. They will retrieve all relevant full-text studies/publications after which two authors will independently screen the full text in order to identify and record reasons for exclusion of the ineligible studies. We will resolve any disagreement through discussion. Trial selection will be displayed in an adapted flow diagram as per the Preferred Reporting Items for Systematic Reviews and Meta-Analyses (PRISMA) statement (Additional file 2).

#### Data extraction and management

Data extraction will be performed independently by at least two authors (PP and RR), who will both compare the extracted data. Disagreements will be resolved by a third author (GA or IL). We will assess duplicate publications and companion papers of a trial together to evaluate all available data simultaneously (maximise data extraction, correct bias assessment). Trial authors will be contacted by email to request any additional data which may not have been reported sufficiently or at all in the publication. Review Manager software will be used to extract data.

### Assessment of risk of bias in included studies

The risk of bias of every included trial will be evaluated independently by at least two authors. In case of any disagreement, discrepancies will be discussed with a third author and resolved by consensus. The risk of bias will be assessed using the Cochrane’s ‘Risk of bias’ assessment tool [52, 53] and the Cochrane Effective Practice and Organisation of Care Group’s guidance [54]. We will evaluate the methodology in respect of the following:

- Random sequence generation
- Allocation concealment
- Blinding of participants and treatment providers
- Blinding of outcome assessment
- Incomplete data outcome
- Selective outcome reporting
- Other risks of bias
- Overall risk of bias

Classification of the trials will follow criteria defined in Additional file 3 [37, 55–60].

### Meta-analysis

Both end scores and change-from-baseline scores will be used to analyse continuous outcomes. If both end scores and change-from-baseline scores are reported, then only end scores will be used. If only change-from-baseline scores are reported, these results together with end scores will be analysed in the same meta-analyses [61]. Exploratory outcomes will be analysed using change from baseline scores.

Data will be meta-analysed by RevMan 5 statistical software [50]. We will use STATA statistical software (STATA 2015) in case of zero event trials, where RevMan 5 zero event handling is insufficient [62, 63].

Intervention effects will be assessed by both random-effects model meta-analyses and fixed-effect model meta-analyses [55, 64, 65], using the more conservative point estimate of the two. Three primary outcomes will be examined with *P* ≤ 0.025 being statistically significant. An eight-step procedure will be used to assess if the thresholds for significance are crossed. Five secondary outcomes will be examined with *P* ≤ 0.017 being statistically significant [48]. The results of the exploratory outcomes will be considered hypothesis generating only.

Analysis of all included studies will be compared to a sensitivity analysis of studies at low risk of bias. If the results are similar, primary conclusions will be based at the time point closest to 12 months on the overall analysis. If the results differ, primary conclusions will be based on studies with a low risk of bias.

A table describing the types of serious adverse events in each trial will be provided.

### Trial Sequential Analysis

Traditional meta-analysis runs the risk of random errors due to sparse data and repetitive testing of accumulating data when updating reviews. Trial Sequential Analysis will thus be used to analyse the outcomes in order to calculate the required information size and control the risks of type I errors and type II errors [37, 56].

For continuous outcomes, Trial Sequential Analysis will use the observed SD, a mean difference of the observed SD/2, an alpha of 2.5% for the three primary outcomes, an alpha of 1.67% for the five secondary outcomes, and a beta of 10%, with adjustment for observed diversity [58, 66]. Mean differences (MDs) and the standardised mean difference will be expressed with 95% confidence intervals (CI) for continuous outcomes, as well as the Trial Sequential Analysis-adjusted CIs for MDs.

For dichotomous outcomes, Trial Sequential Analysis will use the proportion of participants with an outcome in the control group, a relative risk reduction of 20%, an alpha of 2.5% for primary outcomes, an alpha of 1.67% for secondary outcomes, and a beta of 10%, with adjustment for observed diversity [58]. We will calculate risk ratios with 95% CI for dichotomous outcomes, as well as Trial Sequential Analysis-adjusted CIs.

### Subgroup analyses

Subgroup analysis when analysing the primary outcomes will be performed as follows:

- Trials at high risk of bias compared to trials at low risk of bias.
- Trials stratified according to experimental interventions.
- Trials stratified according to control interventions.
- Trials according to use of co-interventions.
- Complexity: trials with participants with no co-morbidities compared to trials with participants pre-existing co-morbidities.
- Trials in which the experimental intervention was evaluated by either the parents or the child after the treatment sessions had been delivered compared to trials in which the experimental intervention was not evaluated by either the parents or the child after the treatment sessions had been delivered.

We will use the formal test for subgroup interactions in Review Manager [50].

### Sensitivity analyses

To assess the potential impact of bias, we will perform a sensitivity analysis to exclude trials at an overall ‘high risk of bias’.

To assess the potential impact of the missing data for dichotomous outcomes, we will perform the following sensitivity analyses.

- ‘Best-worst-case’ scenario: assume that all participants lost to follow-up in the experimental group had no serious adverse events, including not developing any psychiatric disease such as an eating disorder.
- ‘Worst-best-case’ scenario: assume that all participants lost to follow-up in the experimental group, had a serious adverse event, for instance, developing a psychiatric disease such as an eating disorder.

Statistical heterogeneity will be assessed by visual inspection of the forest plots and *I*^2^ statistic values [48]. Underlying reasons behind statistical heterogeneity in meta-analyses will be investigated by assessing trial characteristics.

### Summary of findings table

A summary of findings table using each of the prespecified primary outcomes will be presented using GRADE considerations for studies contributing data to the meta-analyses for the prespecified outcomes [48, 59, 60, 67–78]. Methods and recommendations described in Chapter 8 (Section 8.5) and Chapter 12 of the *Cochrane Handbook for Systematic Reviews of Interventions* will be followed using GRADEpro software [79].

## Discussion

This review aims to provide evidence on the beneficial and harmful effects of smartphone apps as an intervention in children with overweight. Currently, there is no comprehensive systematic review of smartphone interventions in children with overweight to inform clinical practice. Previous systematic reviews in this population have considered the efficacy of mobile health technologies more broadly in the role of weight management [29], but none have provided comprehensive coverage of the benefits and harms of smartphone apps. Hence, this evidence will hopefully help children, their parents, and health professionals to make informed treatment decisions. This review will also highlight any gaps in the evidence base of such interventions which will help to shape the development and optimisation of future potential interventions.

This protocol has several strengths. The predefined methodology is based on the Cochrane Handbook for Systematic Reviews of Interventions and considering the risk of bias, Trial Sequential Analysis, and GRADE assessment [61, 64, 75]. We will assess both experimental and control interventions combined as well as individually, thereby being able to identify why interventions seem to work and under what conditions. This protocol, therefore, takes into account both the risks of systematic errors, the risk of random errors, and the risks of design errors [47].

The primary limitation of our protocol is that we are accepting interventions that have used all subtypes of smartphone apps. Hence, the different types of interventions with apps may have different effects compared with usual care, the statistical heterogeneity might be considerable and meta-analysis of all trials in one analysis might not be valid. A second limitation is the large number of subgroup analyses which increases the risk of a type I error. We have adjusted our thresholds for significance according to the number of primary outcomes, and the risk of type I errors and type II errors will be taken into account when interpreting the results of the review.

## Supplementary information

**Additional file 1.** Preliminary search strategy for MEDLINE (Ovid).

**Additional file 2.** Prisma-P+ checklist.

**Additional file 3.** Classification of randomised trials at low and at high risk of bias.

## Data Availability

Not applicable

## Abbreviations

Apps: Applications
BMI: Body mass index
Cis: Confidence intervals
DEXA: Dual energy X-ray absorptiometry
GRADE: Grading of Recommendations Assessment, Development, and Evaluation
MDs: Mean differences
mHealth: Mobile health
SD: Standard deviation
WHO: World Health Organization
