# Supplementary material for: Health apps targeting children with overweight—a protocol for a systematic review with meta-analysis and Trial Sequential Analysis of randomised clinical trials
Source: Syst Rev. 2020 Feb 11;9:28. doi: 10.1186/s13643-020-1269-0 (PMC7014738; doi:10.1186/s13643-020-1269-0)
Supplement: Supplementary file 1 — Additional file 1. Preliminary search strategy for MEDLINE (Ovid). [file 13643_2020_1269_MOESM1_ESM.docx]

**Additional File 1**

NOTES: unless stated otherwise, search terms are free text terms; MeSH: Medical subject heading (Medline medical index term); an asterisk (*) stands for ’any character(s)’, a question mark stands for ’one or no character’.

1. exp Obesity/

2. exp Hyperphagia/

3. exp body mass index/

4. exp Weight Gain/

5. exp Weight Loss/

6. exp Anti-Obesity Agents/

7. (Pickwick* syndrom* or Prader willi syndrom* or obes* or adipos* or overweight* or 'over weight*' or overeat* or 'over eat*' or 'over feed*' or overfeed* or binge eating disorder* or 'fat overload' syndrom* or (weight and (gain or cycling or reduc* or loss or losing or maint* or decreas* or watch* or diet* or control*))).ti,ab.

8. 1 or 2 or 3 or 4 or 5 or 6 or 7

9. exp Mobile Applications/

10. ((mobile or smartphone or telephone or virtual or digital or wellness or medical or dietary or physical activity or intervention or treatment or weight or calorie) and (app or coach or tracker)).mp. [mp=title, abstract, original title, name of substance word, subject heading word, floating sub-heading word, keyword heading word, protocol supplementary concept word, rare disease supplementary concept word, unique identifier, synonyms]

11. (mhealth or 'in touch' or txt2bfit or pegaso or fitbit or collective intelligence or 'move it' or myfitnesspal).mp. [mp=title, abstract, original title, name of substance word, subject heading word, floating sub-heading word, keyword heading word, protocol supplementary concept word, rare disease supplementary concept word, unique identifier, synonyms]

12. 9 or 10 or 11

13. 8 and 12

14. (random* or blind* or placebo* or meta-analys*).mp. [mp=title, abstract, original title, name of substance word, subject heading word, floating sub-heading word, keyword heading word, protocol supplementary concept word, rare disease supplementary concept word, unique identifier, synonyms]

15. 13 and 14
